# Equipping future nurses: readiness of nursing students in addressing intimate partner violence in China

**DOI:** 10.3389/fpubh.2025.1627062

**Published:** 2025-10-16

**Authors:** Le Chen, Norhasmah Mohd Zain, Raishan Shafini Bakar, Qin Qin, Ershan Xu, Shu Tang, Hongtao Chen, Zhixing Huang, Jing Chen

**Affiliations:** ^1^School of Nursing, Xiangnan University, Chenzhou, Hunan, China; ^2^School of Health Sciences, Universiti Sains Malaysia, Kota Bharu, Kelantan, Malaysia; ^3^School of Medical Sciences, Universiti Sains Malaysia, Kota Bharu, Kelantan, Malaysia; ^4^Department of Medical, Quzhou College of Technology, Quzhou Zhejiang, China; ^5^School of Nursing, Hunan University of Medicine, Huaihua, Hunan, China; ^6^Puai Medical College, Shaoyang University, Shaoyang, Hunan, China; ^7^School of Nursing, Hunan University of Chinese Medicine, Changsha, Hunan, China; ^8^College of Medical Imaging Laboratory and Rehabilitation, Xiangnan University, Chenzhou, Hunan, China

**Keywords:** intimate partner violence, nursing students, readiness to respond, competency-based training, public health education

## Abstract

**Aim:**

This study aimed to evaluate the readiness of nursing students in China to respond to patients experiencing intimate partner violence (IPV) and to identify key influencing factors.

**Background:**

Nurses play a crucial role in providing care and support to IPV survivors. However, limited research has examined the extent to which nursing students are prepared for this responsibility. Gaining insights into their level of readiness and the factors that shape it is essential for informing educational strategies and policy development.

**Design:**

A cross-sectional study was conducted among 532 nursing students recruited from four public universities in Hunan Province, China, via multistage sampling.

**Methods:**

Data were collected using validated instruments, including the Readiness to Encounter Partner Abuse Patients (READI) Scale and the Modified Physician Readiness to Manage Intimate Partner Violence Survey (PREMIS). Pearson correlation analyses were conducted to examine the associations among perceived knowledge, attitudes, skill preparedness, and readiness. Logistic regression (LR) was employed to identify significant predictors of readiness, and mediation analyses were performed to assess the intermediary roles of perceived knowledge, skill preparedness, and attitudes.

**Results:**

82.7% of participants reported poor readiness to respond to IPV cases. Significant predictors of readiness included perceived knowledge (OR=2.938; 95% CI: 2.246–3.931), skill preparedness (OR=3.592; 95% CI: 2.700–4.095), attitudes (OR=4.472; 95% CI: 1.925-10.800), IPV-related education (OR=2.654; 95% CI: 1.297–5.230), and IPV training experience (OR=3.072; 95% CI: 1.444–6.310). Mediation analyses further revealed that both perceived knowledge and skill preparedness partially mediated the relationship between IPV training and readiness.

**Conclusion:**

The study identified a substantial gap in the readiness of nursing students to manage IPV-related cases, underscoring the critical need to enhance educational and training programs. Incorporating comprehensive IPV-related content into nursing curricula may significantly improve preparedness and foster survivor-centered care within the healthcare system.

## Introduction

Intimate partner violence (IPV) is a pervasive form of domestic violence that encompasses physical, sexual, psychological, or controlling behaviors inflicted by an intimate partner, often resulting in significant harm to the victim (1). Recognized as a global public health crisis, IPV is among the leading causes of morbidity and mortality among women (2).

Alarmingly, an estimated 27% of women worldwide experience IPV in their lifetime, with many facing violence early in life, particularly during adolescence and young adulthood (3). In mainland China, the estimated prevalence of IPV ranges from 17.4 to 24.5% for psychological violence, 2.5 to 5.5% for physical violence, and 0.3 to 1.7% for sexual violence (4). Shockingly, IPV is implicated in approximately 40% of homicides and 60% of suicides among women in China (5).

The health consequences of IPV for women are profound and multifaceted, encompassing a spectrum of adverse outcomes. These include mental health disorders such as depression and post-traumatic stress disorder (PTSD), sexual and reproductive health complications including unintended pregnancies and miscarriages, as well as deleterious effects on children, such as behavioral disturbances and perpetuation of intergenerational cycles of violence (6). IPV survivors are also at heightened risk of substance abuse, suicidal ideation, and delivering low birth weight infants. The ripple effects of IPV underline the urgency of effective intervention and support systems.

Healthcare systems play a pivotal role in the identification and management of IPV. Women affected by IPV frequently access healthcare services for conditions related to abuse, often without disclosing the violence itself (7). Healthcare services provide a unique and often critical opportunity to identify IPV survivors, deliver appropriate care, and connect them with further support to prevent ongoing harm (8).

Healthcare professionals often represent the first and most trusted point of contact for IPV survivors (9). Nurses, who constitute the largest segment of the healthcare workforce, are particularly well-positioned to provide person-centered care to IPV survivors (10). In mainland China, more than 4.5 million nurses constitute the frontline of healthcare service provision (11). They are critical in documenting IPV-related diagnoses and treatments, assisting victims in seeking legal recourse, and providing resources and referrals (12). Moreover, under the *Anti-Domestic Violence Law of the People’s Republic of China*, healthcare institutions are mandated to report specific cases of IPV, further reinforcing the critical role of healthcare personnel in addressing this pervasive issue (13).

Given their central role, nurses must be adequately prepared to support IPV survivors. Therefore, nursing students are expected to develop the requisite knowledge, clinical competencies, and appropriate attitudes during their training to fulfill this responsibility as future registered nurses (14). Readiness, defined as a psychological state encompassing the cognitive, motivational, and emotional preparedness to address a specific issue, is a critical factor in enabling effective IPV response (15, 16). Prior evidence has identified several determinants of readiness, including exposure to IPV-related education, targeted training, and perceived knowledge. For instance, extended healthcare education and IPV-specific instruction are positively associated with higher levels of readiness among healthcare providers (17–19). Other studies have shown that knowledge, staff capabilities, and preparation are significant predictors of readiness, while age, perceived knowledge, and attitudes also play important roles (20, 21). Conversely, negative attitudes, inadequate training, and the absence of institutional protocols have been identified as critical barriers to effective IPV response (22).

Although a growing number of empirical studies have explored nursing students’ readiness to address IPV, most of these studies have been conducted in Western or Middle Eastern contexts (23), with limited evidence from China. Furthermore, although existing research has demonstrated that IPV training can improve students’ readiness, few studies have explored the underlying mechanisms through which such training exerts its influence on readiness (23). This study addresses these gaps by investigating the readiness of nursing students in Hunan Province, China, to support IPV survivors. Specifically, it explores the influence of perceived knowledge, skill preparedness, and attitudes on readiness and further examines the mediating roles of perceived knowledge and skill preparedness in the relationship between IPV training and readiness. By shedding light on these factors, this study seeks to inform educational strategies and policy initiatives to enhance the readiness of future nurses to respond effectively to IPV cases.

## Methods

### Study design and participants

A cross-sectional survey was conducted from May to July 2024, targeting undergraduate nursing students enrolled in four public universities in Hunan Province. To ensure the representativeness of the sample, a multistage random sampling strategy was adopted. In the first stage, Hunan Province, which hosts eight public universities offering undergraduate nursing education, was divided into four geographical regions: East (three universities), West (one university), South (two universities), and North (two universities). Except for the western region, which had only one eligible university, one university from each of the remaining regions was randomly selected using computer-generated random numbers with the RAND function in Microsoft Excel: Hunan University of Chinese Medicine (East), Hunan University of Medicine (West), Xiangnan University (South), and Shaoyang University (North).

In the second stage, approximately half of the fourth-year nursing classes (final academic year) from each selected university were randomly sampled. A complete list of eligible classes was obtained from each selected university. Subsequently, simple random sampling was performed by generating random numbers via the RAND function in Excel to select the final sample. Specifically, two classes were randomly selected from both Hunan University of Chinese Medicine and Hunan University of Medicine, three classes from Xiangnan University, and four classes from Shaoyang University. All eligible students within the selected classes were invited to participate in the study. Inclusion criteria were: (1) full-time, fourth-year undergraduate nursing students officially registered at their respective institutions; (2) voluntary participation with provision of written informed consent; (3) age of 18 or older; (4) completion of clinical internship; and (5) successful completion of the College English Test Band 4 (CET-4). International students were excluded from participation. 557 students took part in the survey. After excluding 25 invalid questionnaires, 532 valid responses were retained for the final analysis.

The sample size was calculated based on the single mean formula method in Equation $n={\left(\frac{Z(\mathrm{SD})}{\Delta }\right)}^{2}$ , where ∆= 0.16 [representing the desired precision based on an expected estimation error of 5% and a mean score reported in a previous study (24)]. The sample size was further inflated by 20% to account for possible missing data, giving rise to a total sample size of 423 students. The final sample consisted of 532 participants, and a *post hoc* power analysis conducted via the pwr package in R confirmed a statistical power of 100%.

### Study instruments

The study instrument comprised a self-developed questionnaire and a standardized questionnaire. The research team designed a 9-item general information questionnaire to collect demographic characteristics and information related to IPV. Demographic variables included age, gender, and place of residence. IPV-related items assessed exposure to IPV education within academic curricula, participation in IPV training programs, observation of healthcare professionals managing IPV cases, provision of care to IPV victims, as well as personal experiences with IPV or having witnessed such incidents. The standardized questionnaires consisted of two components: the Readiness to Encounter Partner Abuse Patients (READI) Scale and a questionnaire evaluating nursing students’ knowledge, attitudes, and perceived preparedness in skills related to IPV.

The second part of the questionnaire is on the READI Scale. The READI scale, developed by Sawyer et al. (16), assessed participants’ readiness to address IPV. This 27-item scale comprises four dimensions: self-efficacy (12 items), emotional readiness (5 items), motivational readiness (5 items), and partner abuse knowledge (5 items). Items were rated on a 7-point Likert scale (1 = completely disagree to 7 = completely agree). Scores were categorized as low (1–4.99), moderate (5–5.99), or high readiness (6–7). In this study, a mean score below 5 indicated poor readiness, while a score of 5 or higher reflected good readiness. The original scale demonstrated excellent reliability(Cronbach’s *α* = 0.92) (16). Reliability for this study ranged from 0.714 to 0.904 across dimensions, with an overall Cronbach’s α of 0.846.

The final section of the questionnaire focused on knowledge, attitudes, and preparedness related to IPV. The Modified Physician Readiness to Manage Intimate Partner Violence Survey (PREMIS) (25) was utilized to evaluate participants’ IPV-related knowledge, attitudes, and skill preparedness. In the present study, four subscales from the Modified PREMIS were employed. Reliability analyses were conducted for each subscale as detailed below:

1. Actual Knowledge Scale: this scale assessed objective IPV knowledge through 18 items, including single-choice, multiple-choice, matching, and true/false formats. Scores ranged from 0 to 38, with higher scores indicating greater knowledge. The Kuder–Richardson 20 reliability coefficient was 0.56 in the original study (26) and 0.59 in this study.
2. Perceived Knowledge Scale: this scale measured self-assessed IPV knowledge via 14 items on a 7-point Likert scale (1 = Nothing to 7 = Very Much). Higher scores reflect greater perceived knowledge. The Cronbach’s *α* was 0.96 for both the original (25) and this study.
3. Skill Preparedness Scale: this scale evaluated readiness for IPV-related clinical tasks using 10 items rated on a 7-point Likert scale (1 = Not Prepared to 7 = Quite Well Prepared). Higher scores indicated better skill preparedness. The Cronbach’s *α* was 0.97 for the original study (25) and 0.95 for this study.
4. Attitudes Scale: this scale assessed attitudes toward IPV using 26 items rated on a 7-point Likert scale (1 = Strongly Disagree to 7 = Strongly Agree). Higher scores indicated more positive attitudes. The Cronbach’s *α* for the original scale was 0.51 (26), while in this study, the total Cronbach’s α was 0.45.

The original READI and modified PREMIS scales are available only in English, and no validated Chinese versions currently exist. In the present study, the original English versions were employed, as all participants had passed the CET-4 and were deemed capable of comprehending English-language questionnaires.

Before the formal survey, a pilot test was conducted using a convenience sample of 30 fourth-year undergraduate nursing students from one university. The eligibility criteria for the pilot sample were consistent with those described for the main study. The pilot aimed to assess the clarity and comprehensibility of the questionnaire items. Feedback from the participants indicated that the items were generally clear and easy to understand, and no major revisions were deemed necessary.

To evaluate the structural validity of the scales in the Chinese context, exploratory factor analysis (EFA) was conducted on the study sample. The Kaiser-Meyer-Olkin (KMO) measure for the READI scale was 0.93, and Bartlett’s test of sphericity was significant (*χ*^2^ = 7502.059, *p* < 0.001), with a cumulative variance explained of 51.1%. The overall KMO for the Modified PREMIS scale was 0.91, and Bartlett’s test was likewise significant (*χ*^2^ = 2348.17, *p* < 0.001), yielding a cumulative variance explained of 40.4%. For the subscales, the Perceived Knowledge Scale had a KMO of 0.97 with 66.2% cumulative variance explained; the Skill Preparedness Scale had a KMO of 0.94 with 66.1% cumulative variance explained; and the Attitudes Scale had a KMO of 0.89 with 43.4% cumulative variance explained. All corresponding Bartlett’s tests were statistically significant (*p* < 0.001). These results support the structural validity and applicability of the scales among undergraduate nursing students in China.

### Data collection procedure

Data were collected using the online survey platform Wenjuanxing.^1^ At each participating university, a faculty member from the School of Nursing was designated as the research coordinator and was responsible for disseminating the survey link to eligible students via QQ and WeChat, both of which are widely utilized social media platforms in China. Students could access the questionnaire through either a direct hyperlink or a QR code. Before participation, informed consent was obtained from all respondents, who were explicitly informed of their right to withdraw from the study at any point without penalty. To ensure data completeness, the survey platform required that all items be completed before submission. Furthermore, to uphold data integrity, each participant was permitted to submit only one response. All submitted questionnaires were rigorously reviewed, and invalid responses were excluded from analysis.

### Data analysis

Data was analyzed via R 4.4.1. Continuous variables with normal distributions were reported as mean ± standard deviation (SD) and compared using independent *t*-tests. Categorical variables were reported as *n* (%) and analyzed using chi-square tests. Pearson correlation coefficients assessed relationships among knowledge, attitudes, skill preparedness, and readiness. Multiple logistic regression (LR) identified factors influencing readiness to respond to IPV. Model 1 was unadjusted; Model 2 was adjusted for age, gender, and region of residence. Mediation analysis examined the roles of perceived knowledge, skill preparedness, and attitudes in the relationship between IPV training and readiness. Statistical significance was set at *p* < 0.05.

### Ethical considerations

The study was approved by the Human Research Ethics Committee of Universiti Sains Malaysia (JEPeM Code: USM/JEPeM/KK/24010132) and the Ethics Committee of Xiangnan University (Approval No.: 2023YXLL036). All participants were fully informed of the study’s objectives, procedures, and the measures taken to ensure anonymity and confidentiality. Participants’ rights and autonomy were fully respected, and they were informed of their right to discontinue participation at any time without adverse consequences.

## Results

### Characteristics of participants

This study involved 532 senior undergraduate nursing students, the majority of whom were female (85.0%), with a smaller proportion of male participants. The mean age was 21.93 years (SD = 1.13), and more than half (57.7%) resided in rural areas. Notably, a substantial proportion of students (92.1%) reported receiving no formal instruction on IPV during their nursing education. Only 6.6% had participated in IPV-related training; among them, 6.0% engaged in video-based learning, while 3.9% acquired knowledge through reading materials such as books, newspapers, or magazines.

Awareness of IPV screening and care practices at clinical internship sites was generally limited. Nearly half (46.4%) of the students were uncertain whether healthcare providers at their internship hospitals conducted IPV screening or offered care to women experiencing abuse. Additionally, 30.8% indicated that such practices were not performed at their clinical sites. A significant majority (81.0%) had never provided care to IPV survivors. Furthermore, 10.7% of students disclosed personal experiences of IPV, and 32.5% reported having witnessed IPV among family members. These findings reveal critical deficiencies in IPV education and preparedness among nursing students and underscore the urgent need for comprehensive training and awareness initiatives. Detailed demographic and background characteristics of the participants are presented in Table 1.

**Table 1:** Demographic characteristics of participants (*N* = 532).

Variables	*N* (%)
Age, years (Mean ± SD)	21.93 ± 1.13
Gender	
Male	80 (15.0)
Female	452 (85.0)
Region of living	
Urban	225 (42.3)
Rural	307 (57.7)
Have you been educated about domestic violence (including IPV) in your school course?	
No	490 (92.1)
Yes	42 (7.9)
Have you ever received training on domestic violence (including IPV)?	
No	497 (93.4)
Yes	35 (6.6)
If so, how much training or studying about IPV issues have you had in nursing school?	
Watched a video	32 (6.0)
Attended a lecture or talk	14 (2.6)
Attended a skill-based training or workshop	8 (1.5)
Books, newspapers, magazines	21 (3.9)
Do other health professionals screen or provide nursing care for women for IPV in the hospital or community clinic while you practice?	
Yes	121 (22.7)
No	164 (30.8)
Do not know	247 (46.4)
While you practice in hospitals or community clinics, have you had experience responding to IPV?	
Yes	101 (19.0)
No	431 (81.0)
Personal Experience	
Have you experienced IPV	
Yes	57 (10.7)
No	475 (89.3)
Have you witnessed IPV	
Yes	173 (32.5)
No	359 (67.5)

IPV = intimate partner violence.

### Nursing students’ readiness to respond to IPV

Among the 532 participants, 440 (82.7%) exhibited poor readiness, while 92 (17.3%) demonstrated good readiness. Notably, the “well readiness” category included 85 participants (16.0%) with moderate readiness and 7 participants (1.3%) with high readiness. The detailed readiness levels of the nursing students are presented in Table 2. Poor performance was observed across most domains, particularly in self-efficacy (81.6%), emotional readiness (77.6%), and partner abuse knowledge (70.7%). Only a small proportion showed high readiness in these domains, ranging from 2.6 to 8.1%. However, motivational readiness stood out as the strongest area. 41.7% of students achieved high readiness, 34.2% showed moderate readiness, and only 24.1% exhibited low readiness. These findings underscore the urgent need for targeted interventions to enhance readiness among future healthcare professionals.

**Table 2:** Readiness among nursing students (*N* = 532).

READI score	Mean ± SD	Low readiness no. (%)	Moderate readiness no. (%)	High readiness no. (%)
Self-efficacy	4.20 ± 0.90	434 (81.6)	84 (15.8)	14 (2.6)
Emotional readiness	4.00 ± 1.22	413 (77.6)	76 (14.3)	43 (8.1)
Motivational readiness	5.53 ± 0.94	128 (24.1)	182 (34.2)	222 (41.7)
Partner abuse knowledge	4.35 ± 0.97	376 (70.7)	141 (26.5)	15 (2.8)
Total readiness	4.43 ± 0.60	440 (82.7)	85 (16.0)	7 (1.3)

READI = readiness to encounter partner abuse patients scale; SD = standard deviations.

### Nursing students’ IPV-related knowledge, attitudes, and skill preparedness

The mean score for actual IPV-related knowledge was 19.79 ± 4.22, corresponding to a moderate proficiency level with 52.1% of answers correct. Self-reported measures by students indicated moderate levels of perceived knowledge (mean ± SD: 3.79 ± 1.10), skill preparedness (4.39 ± 1.13), and attitudes (4.03 ± 0.29) (see Supplementary Table S1). These findings underscore the imperative to enhance educational curricula and training programs to elevate students’ IPV management competencies.

### Correlation between knowledge, attitudes, skill preparedness, and readiness

Pearson correlation analysis revealed a positive correlation between readiness, perceived knowledge, actual knowledge, skill preparedness, and attitudes. Total readiness has a strong positive correlation with skill preparedness (*r* = 0.63, *p* < 0.001) and a moderate correlation with perceived knowledge (*r* = 0.55, *p* < 0.001), while its correlation with actual knowledge was weak but significant (*r* = 0.10, *p* = 0.023) and with attitudes was weak to moderate (*r* = 0.25, *p* < 0.001). These results, as presented in Supplementary Table S2, emphasized the importance of fostering these elements to improve readiness.

### Associations between demographic characteristics and readiness

As presented in Table 3, demographic variables, particularly exposure to IPV education within the formal school curriculum and participation in IPV-related training, were significantly associated with enhanced readiness to respond effectively to IPV.

**Table 3:** Associations between demographic characteristics and readiness (*N* = 532).

Variables	Poor readiness	Well readiness	χ^2^	*p* value
Age, years	21.92 ± 1.12	21.97 ± 1.13	−0.381	0.703^b^
Gender			0.731	0.393^a^
Male	63 (14.3)	17 (18.5)		
Female	377 (85.7)	75 (81.5)		
Region of living			0.694	0.405^a^
Urban	182 (41.4)	43 (46.7)		
Rural	258 (58.6)	49 (53.3)		
IPV education in school courses			7.030	0.008^a^*
No	412 (93.6)	78 (84.8)		
Yes	28 (6.4)	14 (15.2)		
IPV training			8.889	0.003^a^*
No	418 (95.0)	79 (85.9)		
Yes	22 (5.0)	13 (14.1)		
Seeing others screen or provide nursing care for women for IPV			3.765	0.152^a^
Yes	93 (21.1)	28 (30.4)		
No	139 (31.6)	25 (27.2)		
Do not know	208 (47.3)	39 (42.4)		
Experience responding to IPV			0.091	0.762^a^
Yes	82 (18.6)	19 (20.7)		
No	358 (81.4)	73 (79.3)		
Experiencing IPV			0.003	0.958^a^
Yes	47 (10.7)	10 (10.9)		
No	393 (89.3)	82 (89.1)		
Witnessed IPV			0.699	0.403^a^
Yes	147 (33.4)	26 (28.3)		
No	293 (66.6)	66 (71.7)		

IPV = intimate partner violence; a = chi-square test, b = independent T-test, *p -value is significant at <0.05.

### Influencing factors for readiness

Univariate LR analyses (Model 1) identified several significant predictors of readiness, including perceived knowledge, skill preparedness, attitudes, IPV education integrated into school curricula, and IPV-related training. These associations remained statistically significant in the multivariate adjusted model (Model 2), which controlled for age, gender, and region of residence. Specifically, elevated perceived knowledge (OR = 2.938; 95% CI: 2.246–3.931; *p* < 0.001), skill preparedness (OR = 3.592; 95% CI: 2.700–4.095; p < 0.001), and positive attitudes (OR = 4.472; 95% CI: 1.925–10.80; p < 0.001) were independently associated with greater readiness to address IPV. Additionally, students who had received IPV education in school (OR = 2.654; 95% CI: 1.297–5.230; *p* = 0.006) or IPV-specific training (OR = 3.072; 95% CI: 1.444–6.310; *p* = 0.003) were more likely to demonstrate higher readiness levels. These findings, summarized in Table 4, affirm the critical role of formal education and practical training in augmenting preparedness.

**Table 4:** Factors associated with readiness to respond to IPV using multiple logistic regression (*N* = 532).

Variable	Model 1			Model 2		
	OR	95%CI	*p* value	AOR	95%CI	*p* value
Perceived Knowledge Score	2.921	2.238, 3.897	<0.001	2.938	2.246, 3.931	<0.001
Skill Preparedness Score	3.587	2.699, 4.890	<0.001	3.592	2.700, 4.095	<0.001
Attitudes Score	4.214	1.824, 10.09	<0.001	4.472	1.925, 10.80	<0.001
IPV education in school courses (ref: no)	2.641	1.298, 5.167	0.006	2.654	1.297, 5.230	0.006
IPV training (ref: no)	3.127	1.477, 6.392	0.002	3.072	1.444, 6.310	0.003

Model 1: Not adjusted. Model 2: adjusted for age, gender, and region of living.

AOR, adjusted odds ratio. 95% CI, 95% confidence interval. Ref, reference group.

IPV = intimate partner violence.

### Mediation analyses

**Figure 1:**
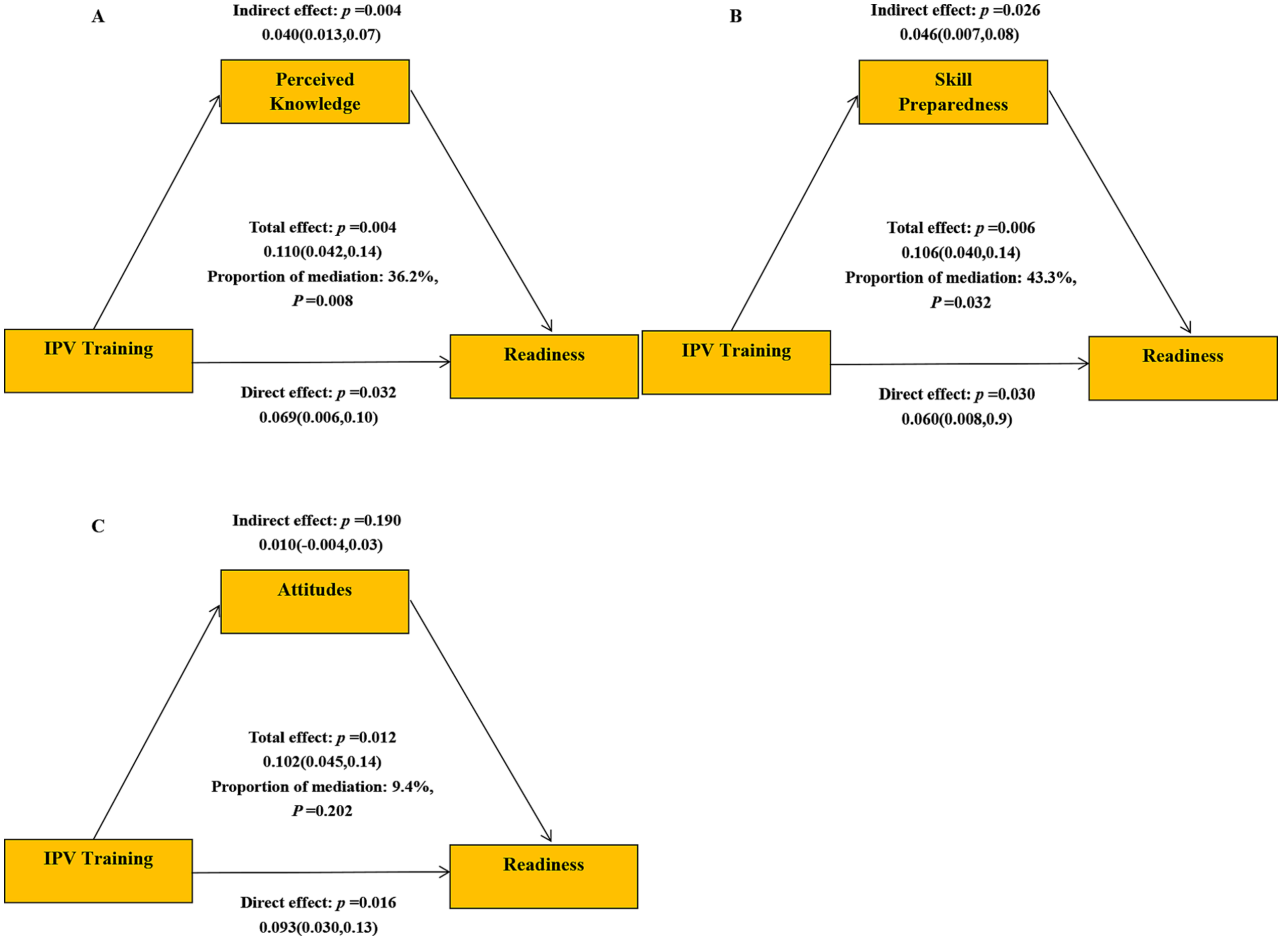
Mediation models of the association between IPV training and readiness. **(A)** Perceived knowledge as mediator. **(B)** Skill preparedness as mediator. **(C)** Attitudes as mediator.

Mediation analyses investigated the pathways through which IPV training influenced readiness, employing perceived knowledge, skill preparedness, and attitudes as mediators (Figure 1). The outcome variable, readiness, was dichotomized (well readiness vs. poor readiness), and mediation was assessed accordingly. Results indicated that IPV training exerted both direct and indirect effects on readiness.

Perceived knowledge mediated the association between IPV training and readiness, with an indirect effect of *β* = 0.040 (95% CI: 0.013–0.07), accounting for 36.2% of the mediation effect (Figure 1A). Skill preparedness also served as a significant mediator, with an indirect effect of β = 0.046 (95% CI: 0.007–0.08), accounting for 43.3% of the mediation effect (Figure 1B). Attitude, however, was not a significant mediator (Figure 1C).

These findings highlighted the critical role of IPV training in enhancing readiness, both directly and by improving perceived knowledge and skill preparedness. This underscored the need for robust IPV-focused training programs to better equip nursing students to respond to IPV patients effectively.

## Discussion

This study sheds light on the readiness of nursing students in China to respond to patients experiencing IPV, providing novel insights into the factors that influence their preparedness. The findings demonstrate that nursing students in China exhibit insufficient preparedness to effectively manage IPV cases. Key determinants influencing their readiness include perceived knowledge, skill competence, attitudinal disposition, and prior exposure to IPV-related education and training. Moreover, the data substantiate the pivotal role of IPV-specific training in enhancing preparedness, with perceived knowledge and skill competence functioning as critical mediators within this framework. These results furnish valuable insights for educators, healthcare practitioners, and policymakers in China, informing the design and advancement of IPV and domestic violence educational programs.

### Lack of IPV education in curricula

The findings reveal a concerning lack of readiness among nursing students in China, with 82.7% categorized as having poor readiness. Notably, readiness scores in this study (mean = 4.43) were lower than those reported among nursing students in Australia (mean = 5.07) and significantly lower than social work students (mean = 5.96) (17). This discrepancy underscores an urgent imperative for tailored educational interventions aimed at bridging the gap in IPV-related training and competency.

The observed low readiness scores are largely attributable to the absence of IPV-specific education within Chinese nursing curricula. Unlike social work students who receive comprehensive, skill-based IPV training (17), nursing students in China often lack exposure to IPV-specific content. This gap leaves them underprepared to identify and manage IPV cases in clinical settings. As previous research highlights (27), the absence of IPV-related education significantly undermines the confidence and competence of healthcare providers, including nursing students, to address IPV effectively.

### Perceived knowledge and skill preparedness as key influencers

This study corroborates the pivotal role of perceived knowledge in shaping nursing students’ readiness to respond to IPV. Those with elevated perceived knowledge exhibit heightened confidence in recognizing IPV cases, managing patient interactions, and repudiating IPV as a social norm. Lidiya Teshome et al. (22) posit that perceived knowledge constitutes a foundational prerequisite for IPV management readiness, suggesting that students with higher perceived knowledge possess a more profound understanding of IPV’s concept, characteristics, and consequences. Such students are more inclined to regard their knowledge base as sufficient for IPV identification and intervention in clinical practice, thereby enhancing their confidence in patient engagement. Furthermore, nursing students who perceive themselves as knowledgeable tend to reject IPV and embrace their professional responsibility in its management (28). Interestingly, actual knowledge did not emerge as a significant factor, suggesting that educational interventions should prioritize practical application over rote learning.

Skill preparedness was also identified as a significant factor influencing readiness. Students reported challenges in key areas such as IPV inquiry, identifying signs of abuse, and developing safety plans, resulting in an overall mean skill preparedness score of 4.39. These findings align with previous studies (29, 30), which underscores the necessity of competency-based training to facilitate the translation of theoretical knowledge into clinical practice. Qualitative data from three mixed-method studies (29, 31, 32) highlight that nursing students perceive their insufficient communication skills as a critical barrier to effective patient care, particularly in managing IPV cases. This perceived deficiency contributes to a sense of unpreparedness, underscoring the pivotal role of skill preparedness in shaping their confidence and readiness for clinical practice. The World Health Organization (WHO) recommends a competency-driven curriculum to ensure healthcare students acquire practical skills in managing IPV cases effectively (33).

### Importance of attitudes

Positive attitudes toward IPV management were strongly correlated with readiness. Students exhibiting supportive and empathetic dispositions were more inclined to engage in IPV screening and to provide appropriate care to victims. Conversely, students harboring negative attitudes tended to blame victims or regard IPV as a private matter, thereby obstructing their willingness to intervene (34, 35). Educational programs must therefore incorporate strategies to shape students’ attitudes, such as survivor testimonials and empathy-building exercises, to foster a supportive approach to IPV care.

Cultural norms and societal attitudes in China critically shape nursing students’ perspectives on IPV. Traditional values emphasizing familial harmony and conflict avoidance may perpetuate perceptions of IPV as a private issue, discouraging intervention (36). Additionally, hierarchical relationships in Chinese society and the influence of authority figures (37, 38), including educators and senior healthcare professionals, may impact students’ willingness to challenge IPV-related stigma. To effectively shape students’ attitudes, educational programs must incorporate culturally relevant strategies, such as discussions on how cultural beliefs influence IPV perceptions and responses. Addressing these culture-specific barriers may enhance the cultivation of supportive and proactive attitudes toward IPV management among nursing students.

### Impact of IPV training or education in school courses

The present findings accentuate the transformative effect of IPV training on readiness. Nursing students exposed to IPV training demonstrated significantly higher levels of perceived knowledge and skill preparedness, which in turn bolstered their readiness to respond. Mediation analyses identified perceived knowledge and skill preparedness as critical mediators, with IPV training exerting substantial indirect effects on readiness.

Despite the benefits, 90% of participants in this study reported no prior IPV training or education, highlighting a critical gap in nursing curricula. A likely primary reason for this is that undergraduate nursing curricula typically do not include IPV training as part of the nursing program (29). Leah Kirk et al. emphasize that integrating IPV training is crucial for helping nursing students incorporate the knowledge they acquire into practice, thereby building their skills and confidence in managing IPV cases (39). Prior research has reported that adding content on domestic violence to curricula positively influences students’ attitudes, their perceptions of the nursing role, and their confidence in managing IPV cases (23, 40, 41). Within the Chinese context, embedding IPV-related content into extant courses, such as community health nursing or medical ethics, may represent a feasible and cost-effective approach. Supplementary IPV education via extracurricular seminars or specialized workshops during clinical internships could further reinforce formal instruction. Interactive pedagogical methods, including role-playing, standardized patient simulations, and repeated IPV scenario exposures, are recommended to enhance students’ perceived knowledge and practical skills (33).

### Strengths and limitations

This study represents a pioneering effort to explore the readiness of nursing students in Hunan Province, China, to respond to IPV and examine the factors influencing their preparedness. This is the first study of its kind conducted in China, offering valuable insights into an underexplored area of nursing education.

One of the key strengths of this study is its methodological rigor, including adherence to random sampling principles and the inclusion of a geographically diverse sample within Hunan Province. This diversity enhances the application of the findings within the province and addresses an important gap in the scientific literature on IPV education in China. Additionally, the findings provide a strong foundation for the development of interventions aimed at improving IPV-related education for nursing students.

However, several limitations must be considered. First, the use of self-reported measures for data collection introduces the possibility of social desirability bias. Although the anonymity of online surveys likely mitigated some of this bias, the influence of reporting bias cannot be entirely ruled out. Moreover, the survey was conducted using an online voluntary participation model, which may have introduced self-selection bias; students with greater interest in the topic of IPV may have been more likely to participate, thereby potentially limiting the application of the findings to the broader student population. To examine the presence of common method bias, Harman’s single-factor test was conducted by entering all items from the main study variables into an exploratory factor analysis. The results indicated that the first unrotated factor accounted for 19.31% of the total variance, markedly below the conventional threshold of 40%, thereby suggesting that common method bias was not a substantial concern in this investigation. Nonetheless, the involvement of nursing faculty members in administering the surveys may have inadvertently influenced student responses, potentially introducing additional bias.

Second, the original English versions of the READI and Modified PREMIS instruments were used, and validated Chinese translations are not yet available. Although all participants had passed the CET-4, and the pilot testing confirmed the general comprehensibility of the items, and exploratory factor analysis supported the structural validity, some items may still have been subject to misinterpretation within the Chinese linguistic and cultural context. Future studies should take into account formal translation and cross-cultural validation of these instruments.

Third, the cross-sectional design of this study precludes causal inferences, as it only allows for the identification of correlations and predictive relationships between variables. Future research should consider employing longitudinal designs or intervention trials to better understand causal pathways and the long-term effects of IPV-related education and training.

Fourth, although intraclass correlation coefficients (ICC) were not explicitly accounted for in the analysis, thus potentially neglecting clustering effects at the university or class level, the *post hoc* power analysis demonstrated that the sample size was sufficient to achieve 100% statistical power, supporting the robustness of the study findings.

Fifth, while the sample was drawn from nursing schools across different regions within Hunan Province, it may not fully represent nursing students in other provinces or regions of China. Expanding the geographical scope in future studies would provide a more comprehensive understanding of IPV readiness among nursing students nationwide.

Despite these limitations, the study makes a significant contribution to the understanding of nursing students’ readiness to address IPV and highlights critical areas for educational reform.

## Conclusion

This study reveals that nursing students in China perceive themselves as unprepared to respond effectively to patients experiencing IPV. Readiness was found to be significantly associated with perceived knowledge, skill preparedness, attitudes, IPV-related education within school courses, and participation in IPV training. Notably, training emerged as a pivotal factor, with perceived knowledge and skill preparedness serving as key mediators in enhancing readiness.

These findings highlight an urgent imperative to incorporate IPV education as a strategic priority within national nursing education policy frameworks. By integrating customized IPV educational modules or implementing focused short-term training programs, nursing educators can substantially augment students’ preparedness to manage IPV cases. Such educational interventions should emphasize competency-based pedagogy, prioritizing practical application and the cultivation of supportive attitudes toward IPV survivors.

This study offers foundational evidence to inform the formulation of future public health education policies and intervention strategies in China. Longitudinal research and interventional trials are warranted to validate these results and to evaluate the efficacy of targeted educational initiatives. By prioritizing IPV education, policymakers and educators can ensure that the forthcoming cadre of Chinese nurses is optimally equipped to address IPV, thereby contributing to improved clinical and psychosocial outcomes for survivors.

### Implications

To enhance nursing students’ readiness to address IPV, a coordinated approach integrating education, policy, and health system reform is essential. This study identified perceived knowledge and skill preparedness as key mediators between IPV training and readiness, underscoring the need for evidence-based, competency-driven educational strategies.

First, policymakers and nursing education authorities should integrate IPV education modules into national nursing curriculum standards. These modules should incorporate structured, skills-based training approaches, such as patient simulations, role-playing, and group case discussions, aligned with public health competencies in violence prevention and survivor-centered care.

Second, training should incorporate culturally sensitive materials, including survivor stories and reflective exercises, to enhance students’ empathy and challenge current societal beliefs that conceal and stigmatize IPV.

Finally, health education policies should promote the routine evaluation of IPV-related training effectiveness and ensure sustained funding for faculty development and teaching resources. Institutionalizing IPV education within the broader public health and health professional training agenda will better equip nursing students to serve as frontline responders and provide more effective support and care for survivors.

## Data Availability

The raw data supporting the conclusions of this article will be made available by the authors, without undue reservation.

## Footnotes

^1^ https://www.wjx.cn/
