# Supplementary material for: Equipping future nurses: readiness of nursing students in addressing intimate partner violence in China
Source: Front Public Health. 2025 Oct 16;13:1627062. doi: 10.3389/fpubh.2025.1627062 (PMC12571759; doi:10.3389/fpubh.2025.1627062)
Supplement: Supplementary file 1 [file Table_1.docx]

**Table S1 Knowledge, attitudes, and skill preparedness among nursing students (N = 532)**

| **Variables** | **Mean ± SD** | **Range** |
| --- | --- | --- |
| Actual Knowledge | 19.79 **±** 4.22 | 0-38 |
| Perceived Knowledge | 3.79 **±** 1.10 | 1-7 |
| Skill preparedness | 4.39 **±** 1.13 | 1-7 |
| Attitudes | 4.03 **±** 0.29 | 1-7 |

SD = standard deviations
