# Supplementary material for: Equipping future nurses: readiness of nursing students in addressing intimate partner violence in China
Source: Front Public Health. 2025 Oct 16;13:1627062. doi: 10.3389/fpubh.2025.1627062 (PMC12571759; doi:10.3389/fpubh.2025.1627062)
Supplement: Supplementary file 2 [file Table_2.docx]

**Table S2 Correlation coefficients between knowledge, attitudes, skill preparedness, and readiness (N = 532)**

|  | Total readiness | | Perceived Knowledge | | Actual Knowledge | | Skill preparedness | |
| --- | --- | --- | --- | --- | --- | --- | --- | --- |
|  | r | *p* value | r | *p* value | r | *p* value | r | *p* value |
| Total readiness | **1** |  |  |  |  |  |  |  |
| Perceived Knowledge | 0.55 | <0.001* | **1** |  |  |  |  |  |
| Actual Knowledge | 0.10 | 0.023* | -0.07 | 0.090 | **1** |  |  |  |
| Skill preparedness | 0.63 | <0.001* | 0.67 | <0.001* | 0.03 | 0.514 | **1** |  |
| Attitudes | 0.25 | <0.001* | 0.13 | 0.002* | 0.22 | <0.001* | 0.18 | <0.001* |

r=correlation coefficient, **p* -value is significant at <0.05
